# Individual differences in executive control relate to metaphor processing: an eye movement study of sentence reading

**DOI:** 10.3389/fnhum.2014.01057

**Published:** 2015-01-13

**Authors:** Georgie Columbus, Naveed A. Sheikh, Marilena Côté-Lecaldare, Katja Häuser, Shari R. Baum, Debra Titone

**Affiliations:** ^1^Department of Psychology, McGill UniversityMontreal, QC, Canada; ^2^Centre for Research on Brain, Language, and Music, McGill UniversityMontreal, QC, Canada; ^3^School of Communication Sciences and Disorders, McGill UniversityMontreal, QC, Canada

**Keywords:** metaphor, idioms, executive control, eye movements, sentence reading, context

## Abstract

Metaphors are common elements of language that allow us to creatively stretch the limits of word meaning. However, metaphors vary in their degree of novelty, which determines whether people must create new meanings on-line or retrieve previously known metaphorical meanings from memory. Such variations affect the degree to which general cognitive capacities such as executive control are required for successful comprehension. We investigated whether individual differences in executive control relate to metaphor processing using eye movement measures of reading. Thirty-nine participants read sentences including metaphors or idioms, another form of figurative language that is more likely to rely on meaning retrieval. They also completed the AX-CPT, a domain-general executive control task. In Experiment 1, we examined sentences containing metaphorical or literal uses of verbs, presented with or without prior context. In Experiment 2, we examined sentences containing idioms or literal phrases for the same participants to determine whether the link to executive control was qualitatively similar or different to Experiment 1. When metaphors were low familiar, all people read verbs used as metaphors more slowly than verbs used literally (this difference was smaller for high familiar metaphors). Executive control capacity modulated this pattern in that high executive control readers spent more time reading verbs when a prior context forced a particular interpretation (metaphorical or literal), and they had faster total metaphor reading times when there was a prior context. Interestingly, executive control did not relate to idiom processing for the same readers. Here, all readers had faster total reading times for high familiar idioms than literal phrases. Thus, executive control relates to metaphor but not idiom processing for these readers, and for the particular metaphor and idiom reading manipulations presented.

## Introduction

Many instances of language incorporate metaphorical uses of words, some of which are familiar but some of which are unfamiliar. Consider *The students grasped the concept*, where the verb *grasp* refers to taking hold of something conceptually rather than physically (its literal interpretation). Such common metaphors may generally go unnoticed. Indeed, when familiarity is high, comprehension may simply proceed by retrieving this familiar metaphoric meaning from memory, in the same way comprehension normally proceeds for other types of figurative language, such as idioms (e.g., Libben and Titone, 2008; Titone et al., 2014). In contrast, consider *The textbooks snored on the desk* where *snore* means “to go unused,” which is metaphorically related to its typical literal meaning, “the sound one makes when one is asleep.” Here, the metaphorical meaning is not as familiar as it was for *grasp*, thus, the mental effort required to comprehend this sentence may increase because its intended metaphorical meaning must be generated in the moment (Kintsch, 2000; Kazmerski et al., 2003; Cardillo et al., 2012).

In this study, we investigated whether individual differences in general cognitive capacities, specifically domain-general executive control, relate to metaphor processing. Moreover, we examined this relationship as a function of metaphor familiarity and other factors relevant to on-line comprehension, such as prior contextual constraint, which may or may not force a metaphorical interpretation. We also investigated, for the same participants, whether a relationship between executive control and comprehension extends to another class of figurative language, idioms, which are likely to be more lexicalized than metaphors, and thus amenable to rapid retrieval from memory. As will be seen, our main conclusion is that individual differences in executive control are indeed important for metaphor processing, in a way that varies with familiarity and prior contextual support, and that potentially differs from idioms.

Most psycholinguistic studies of metaphor have investigated nominal metaphors, such as *My lawyer is a shark*, for which some semantic features of the vehicle *shark* (e.g., viciousness) but not others (e.g., marine animal) are attributed to the topic *lawyer*. While it is debated whether this process occurs through category attribution (Glucksberg, 2001, 2003), shared feature comparison (Bowdle and Gentner, 2005), or feature attribution via a multidimensional semantic network search (Kintsch, 2000, 2001; Kintsch and Bowles, 2002), virtually all agree that metaphor understanding depends on the accumulation of one's past experience with particular metaphoric forms—that is, their familiarity.

Indeed, all theoretical accounts would posit that metaphor comprehension should be faster and more accurate when metaphors are familiar when they are unfamiliar. In the category attribution view (Glucksberg, 2001, 2003), this would arise because people directly retrieve familiar metaphoric features, and easily suppress irrelevant features. In the feature alignment view (Bowdle and Gentner, 2005), this would arise because familiar senses of metaphors tend to become integrated over time with the literal word (see also Kintsch, 2000). Consistent with these views, the figurative meanings of familiar vs. unfamiliar metaphors are primed more quickly (Blasko and Connine, 1993), are judged more quickly in phrasal classification tasks (Mashal and Faust, 2009; Goldstein et al., 2012), and undergo a less computationally intensive comparison process (Goldstein et al., 2012; Lai and Curran, 2013; Mashal, 2013). This leads to faster reading and reaction times (Blasko and Connine, 1993; Blasko and Briihl, 1997; Mashal and Faust, 2009; Lai and Curran, 2013), and increased accuracy (Goldstein et al., 2012; Mashal, 2013).

Moreover, some researchers have emphasized how metaphorical knowledge evolves over time by examining, for example, how unfamiliar metaphors can experimentally be made more familiar through repeated exposure (Cardillo et al., 2012; Goldstein et al., 2012). Similarly, some have posited that the figurative meanings of familiar metaphors become lexicalized over time (i.e., they turn into “dead” metaphors, Bowdle and Gentner, 2005), with their component words becoming more polysemous as familiarity increases over time (Glucksberg, 2001, 2003). For these reasons, our understanding of metaphor processing may relate to other work on single word polysemy or homonymy (e.g., Rayner and Frazier, 1989; Frazier and Rayner, 1990; Frisson and Pickering, 1999; Pickering and Frisson, 2001; Klepousniotou et al., 2008), where the crucial question is how selection occurs when multiple meanings are activated.

Of note, several studies have suggested that resolving lexical ambiguity requires increased executive or cognitive control compared to what is required for comprehending unambiguous words (e.g., Gernsbacher and Faust, 1991; Miyake et al., 1994; Gernsbacher and Robertson, 1995; Wagner and Gunter, 2004). Executive control refers to the cognitive skills that govern planning, working memory, and selective attention (Miyake et al., 2000; Karbach and Kray, 2009), which are thought to rely on intact frontal lobe function (e.g., Miyake et al., 2000; Braver et al., 2001). Gernsbacher and Faust (1991; see also Gernsbacher et al., 1990) showed that readers with low comprehension skill (a potential proxy for low executive control) were less capable of inhibiting inappropriate interpretations of lexically ambiguous words (e.g., deciding that *ace* is not related to *He dug with a spade*). Similar results were found in other work for comprehenders with low reading spans, often taken as a measure of working memory (Gunter et al., 2003; Wagner and Gunter, 2004). For example, Miyake et al. (1994) found that readers with low reading spans took longer to read late-occurring disambiguating contexts when the interpretation was unfamiliar or unexpected, suggesting that working memory was necessary to keep both interpretations active until later disambiguating information arrived. These studies are noteworthy in highlighting how a biased context can change what may be considered optimal within a particular comprehension situation. Accordingly, when a prior context is unbiased, the optimal comprehension strategy might be to maintain activation of multiple word meanings or senses in working memory until subsequent disambiguating information arrives. In contrast, when a prior context is biased, the optimal comprehension strategy might be to immediately select or commit to the contextually relevant interpretation of a word's meaning or sense (e.g., Frazier and Rayner, 1990; Frisson and Pickering, 1999).

Given the potential relation between general cognitive capacities and single-word ambiguity resolution, it is reasonable to expect that executive control should also be important for metaphor processing, and indeed, the literature provides some support for this hypothesis. With respect to metaphors and executive control specifically, Chiappe and Chiappe (2007) showed that people with better inhibitory skills (measured by reverse digit span) produced more accurate metaphor interpretations than those with lower skills. Similarly, Kazmerski et al. (2003) found that high-IQ participants (where IQ was correlated with both working memory and vocabulary performance) were more likely to automatically compute metaphorical meanings than low-IQ participants. High-IQ participants also gave better interpretations for metaphors in a subsequent task. In a neuroimaging study, Prat et al. (2012) found that individuals with low vocabulary and working memory performance showed greater activation in the right inferior and middle frontal gyri when processing nominal metaphors (e.g., *He is a prince*), especially those in biased vs. neutral contexts. These findings cohere with other evidence showing that individuals with executive control deficits (e.g., people with schizophrenia) have difficulties processing metaphors (e.g., Mashal et al., 2013).

The role of executive control may be especially important for unfamiliar metaphors. Consistent with this idea, Mashal et al. (2007) found that unfamiliar two-word metaphors (e.g., *sweet sleep*) led to greater neural activation in frontal brain regions (the left middle frontal gyrus, right inferior frontal gyrus, and right posterior superior temporal sulcus) compared to familiar metaphors and literal phrases. In another study, Mashal (2013) found that people with larger reverse digit spans had better recall, comprehension, and recognition for unfamiliar metaphors compared to unrelated word pairs. Such results are consistent with those of Gernsbacher and Robertson (1995) and Gernsbacher et al. (2001), showing that the need to actively suppress metaphor-irrelevant features in a behavioral task was critical for comprehension.

Thus, several sources of evidence suggest that executive control demands during metaphor processing should differ as a function of familiarity and prior context, however, several questions remain. First, while prior work has investigated how individual differences relate to metaphor processing, this work often conflates more than one kind of individual difference simultaneously (e.g., reading span tasks, where performance is based on language processing, vocabulary knowledge, and working memory capacity). Indeed, the majority of tests in the literature have been verbal and language-based in nature (e.g., reading span), thus making it unclear whether domain-general aspects of executive control relate to performance. Second, although previous work has shown that executive control is necessary for resolving lexical ambiguity, to our knowledge no study has investigated how both familiarity and context jointly influence executive control demands during metaphor processing. Finally, past studies have investigated metaphors presented in isolation (e.g., Mashal et al., 2007; Mashal and Faust, 2009; Goldstein et al., 2012; Mashal, 2013) or used secondary tasks, which could compromise the naturalness of comprehension (e.g., Kazmerski et al., 2003; Pierce et al., 2010; Goldstein et al., 2012; Lai and Curran, 2013).

The present study thus addresses some of these limitations in a sentence reading experiment where participants' eye movements are recorded as they naturally read sentences containing metaphors. With respect to assessing individual differences in executive control, we used a well-studied domain-general executive control task (AX-CPT, e.g., Braver et al., 2001). Specifically, we examined different aspects of the eye movement record to determine exactly when executive control is necessary for computing a metaphorical meaning during the time course of reading, and how that varies as a function of the familiarity of the metaphors in question, and of the degree of contextual support provided by the sentence.

To these ends, in Experiment 1, we created sentences containing metaphors that hinged on a metaphoric interpretation of individual verbs (i.e., predicate metaphors), as well as literal sentences using the same verbs. All sentences had the same structure consisting of subject noun, verb, disambiguating context and neutral ending (e.g., *The textbook snored on the desk at the end of the day*). Each sentence could also have a context word prior to the subject noun that either biased a metaphorical or literal interpretation of the verb (e.g., *The unopened textbooks snored on the desk at the end of the day*). Our eye movement measures assessed how long people read the critical verb region in terms of first pass reading, and the whole metaphor region (i.e., noun + verb) in terms of total reading time, thus incorporating fixations occurring after readers had encountered a later disambiguating context (which should indicate whether the verb should have been interpreted metaphorically or literally). This allowed us to construct a time-course ranging from early to late, enabling us to assess whether individual differences in executive control differentially related to different points of this time-course.

When there was no prior context, we expected that readers would delay committing to a metaphorical interpretation of the verb at the point of the verb (e.g., *snored*), as has been found in prior work on polysemous verbs (Pickering and Frisson, 2001). However, we generally expected that a prior context that biased a specific interpretation of the verb (e.g., *unopened textbooks*) would cause readers to commit to the contextually appropriate interpretation. Of note, we expected that these general effects would be modulated by both metaphor familiarity and individual differences in executive control.

In Experiment 2, we extended the results of Experiment 1 by examining another class of figurative language that is likely to be more lexicalized or familiar than metaphors—idiomatic expressions. Idioms have whole meanings that go beyond the combination of the literal meanings (i.e., *kick the bucket* is not related to the act of kicking nor to a pail), and can be accessed from memory as a single lexical item, while also activating the lexical meanings of the component words (Libben and Titone, 2008). Idioms therefore have meaning ambiguity at the word level, like metaphors, but occur in more predictable word configurations than metaphors, thus functioning to a greater extent than metaphors as highly familiar lexicalized entities. Thus, we generally expected that idioms, unlike metaphors, would not show a strong relation to individual differences in executive control.

## Experiment 1: metaphor processing and executive control

### Method

#### Participants

Thirty-six native speakers of English participated for course credit or compensation of $10/h. All participants were from the McGill or Montreal community, had normal or corrected-to-normal vision and no self-reported history of speech or hearing disorders with a mean age of 22.74 (*SD* = 2.73) and a mean of 16.3 years of education (*SD* = 2.0).

#### Stimuli

We created sentences containing metaphors and literal sentences of the type described above, e.g., *The textbook snored on the desk at the end of the day; The sailor snored in the hammock at the end of the day; The unopened textbooks snored on the desk at the end of the day; The tired sailor snored in the hammock at the end of the day*.

The stimulus set consisted of 256 sentences, which included 64 unique verbs, taken from a larger set of metaphors developed by Cardillo et al. (2010). Cardillo et al. normed these verbs in their metaphorical or literal sentences for literalness, figurativeness, plausibility, naturalness, imageability, frequency and interpretability. We modified the Cardillo et al. sentences by adding a neutral continuation (e.g., *The textbook snored on the desk at the end of the day*). This ensured that neither the verb nor the disambiguating context region was sentence-final. We also modified the sentences by presenting them in two conditions: With an adjective providing context prior to the topic noun of the sentence, or without a prior context (see Table 1).

**Table 1:** **Example sentences from metaphor, literal and with or without modifier conditions**.

**Condition**	**Sentence**
Metaphor—Low familiar	*The textbooks snored on the desk at the end of the day*
Metaphor with context—Low familiar	*The unopened textbooks snored on the desk at the end of the day*
Literal—Low familiar	*The sailor snored in the hammock at the end of the day*
Literal with context—Low familiar	*The tired sailor snored in the hammock at the end of the day*
Metaphor—High familiar	*The model flitted between hair colors all the time*
Metaphor with context—High familiar	*The fickle model flitted between hair colors all the time*
Literal—High familiar	*The butterfly flitted between flower blossoms all the time*
Literal with context—High familiar	*The acrobatic butterfly flitted between flower blossoms all the time*

Because we modified the original Cardillo et al. (2010) sentences, we conducted our own normative study to assess familiarity of the literal and metaphorical uses of the verbs. We asked 23 native English speakers (none of whom participated in the sentence reading task) to rate how familiar the verb was on a seven-point Likert scale (1 = *Not at all familiar*, 7 = *Very highly familiar*) for its use (literal or metaphor) in its specific context. The surveys contained sentences for the full set of 64 verbs, but were divided into two versions so that participants only rated either the literal or metaphorical use of each verb. Each survey was presented in one of two pseudo-randomly ordered lists. Thus, 12 participants rated one version, and 11 participants rated a second version. We then calculated a global familiarity score for each verb by creating a ratio of average metaphoric to literal ratings. Across items, this ratio ranged from 0.71 to 1.19 (mean = 0.98), where a value of 1 indicated that the metaphorical and literal sense of the verb were equally familiar, values greater than 1 indicated that the metaphorical sense was more familiar than the literal sense, and values less than 1 indicated that the metaphorical sense was less familiar than the literal sense. This ratio allowed us to determine relative metaphor vs. literal familiarity.

#### Apparatus

We used an Eye-Link 1000 tower mounted system (SR-Research™, Ontario, Canada) that sampled eye position every millisecond. Viewing was binocular but eye movements were recorded from the right eye only, using a head rest. Stimuli were presented on a 21″ ViewSonic CRT monitor with a screen resolution of 1024 × 768 pixels, using EyeTrack 7.10 software developed at UMass Amherst (blogs.umass.edu/eyelab/software). Text was presented on a single line in yellow 10-point Monaco font on a black background. Three characters subtended approximately 1° of visual angle.

#### Procedure

The research was carried out with the approval of the McGill University Research Ethics Board. Participants completed a language background questionnaire before the reading task. Eye movements were calibrated using a nine-point grid. The verb-context pairings were presented once in each of six counterbalanced lists, such that if a participant viewed the metaphor *The textbook snored on the desk at the end of the day*, s/he would not see the same sentence with the added adjective (*The unopened textbook snored on the desk at the end of the day);* nor would s/he see the literal counterparts of the metaphor stimuli of their list (*The [tired] sailor snored in the hammock at the end of the day*). No participant saw the same metaphor or literal sentence more than once.

In addition to the experimental sentences, participants also read 16 practice sentences, for a total of 80 stimulus sentences in each list, and 54 trials belonging to a second experiment (see Experiment 2). All stimuli were randomly presented. Practice sentences could be figurative or literal. Eight occurred at the beginning of the reading task and eight occurred after a rest break at the midway point. Twenty-two percent of trials were followed by yes-no comprehension questions.

After the main sentence reading task, participants completed an executive control task consisting of the AX-CPT task (Braver et al., 2001). This task uses letter stimuli, but as they are symbolic and not dependent on language processing, the task is domain-general. In this task, participants saw letters one at a time in the center of the screen, and were instructed to press one button when an “X” immediately followed an “A,” and to press another button for all other trials. “AX” target trials occurred in 70% of all trials (total trials = 430), and the remaining 30% of trials were comprised of each of three non-target letter combinations (10% each). Thus, the easiest non-target condition was “BY,” which provides a baseline for comparison of the other non-target trials. Here, “B” stands for any letter which is not “A,” and “Y” stands for any letter that is not “X.” Our measure of interest was based on the “BX” trials because encountering the “X” for these trials would trigger a pre-potent tendency to push the button indicated for target “AX” responses rather than non-target responses. This difficulty is thought of as reactive control. Because of the *a priori* similarity between the processes involved in reactive control and what we expect to be required during metaphor interpretation (i.e., suppressing a pre-potent tendency to interpret the words of a metaphor literally), we derived a cost score for each participant based on the millisecond difference between the average correct reaction times for BY from the average correct reaction times for BX.

### Results

Overall comprehension question accuracy was 96.4%, indicating that participants performed the language task well. Eye movement data were analyzed using linear mixed effects (LME) models (lme4 package, version 0.999999-2; Bates et al., 2013, in the R Project for Statistical Computing environment, version 3.0.2; R Development Core Team, 2013). One important reason for using LME over traditional statistics is that it allows us to investigate continuous variables that are based on subject-related differences (e.g., executive control costs) and item-related differences (e.g., familiarity ratings for metaphors or idioms). This kind of analysis cannot be easily accomplished using traditional ANOVA (see Baayen et al., 2008, for a more detailed account of the rationale for using LME). To index early cognitive processes, such as lexical access, and later cognitive processes, such as semantic integration (Rayner, 1998, 2009; Rayner et al., 2012), we analyzed gaze duration (the sum of all fixation durations during the first pass) of the verb (Verb GD), and total reading time (the sum of all fixation durations) of the whole metaphor region (Metaphor TRT), respectively. Thus, for the sentence *The [unopened] textbook snored on the desk at the end of the day*, we analyzed Verb GD for *snored*, and Metaphor TRT for *textbook snored*.

We fit LME models to each eye movement measure. In each model, familiarity (i.e., metaphor/literal familiarity ratio; continuous), executive control (continuous), context (with or without prior context), and condition (metaphor or literal) were fixed effects. Categorical predictors were deviation coded except where noted otherwise, and all continuous predictors were scaled to reduce collinearity. Maximum correlations among main effects were <0.16 for each main model. Subjects and items (sentences) were random intercepts across the models; random slopes were included in models only when they were statistically warranted (cf. Baayen et al., 2008; Barr et al., 2013). In addition, for consistency across models, we computed *p*-values using the number of model terms minus one for the degrees of freedom. All model formulae were near-identical in that they included a four-way interaction term for familiarity ratio ^*^executive control^*^context^*^condition. For ease of data interpretation, we present the means and standard deviations for all continuous factors in Table 2, with familiarity ratio and executive control median split (recall, they were treated as continuous variables in all models).

**Table 2:** **Means and standard deviations for median split familiarity and executive function in Experiment 1**.

**Variable**	**Mean**	**Min**	**Max**	**SD**
Low familiarity (metaphor vs. literal use ratio)	0.93	0.71	0.98	0.06
High familiarity (metaphor vs. literal use ratio)	1.03	0.98	1.19	0.04
Low executive control (cost score in ms)	119	35	339	83
High executive control (cost score in ms)	−19	−105	31	41

#### Verb GD

**Figure 1:**
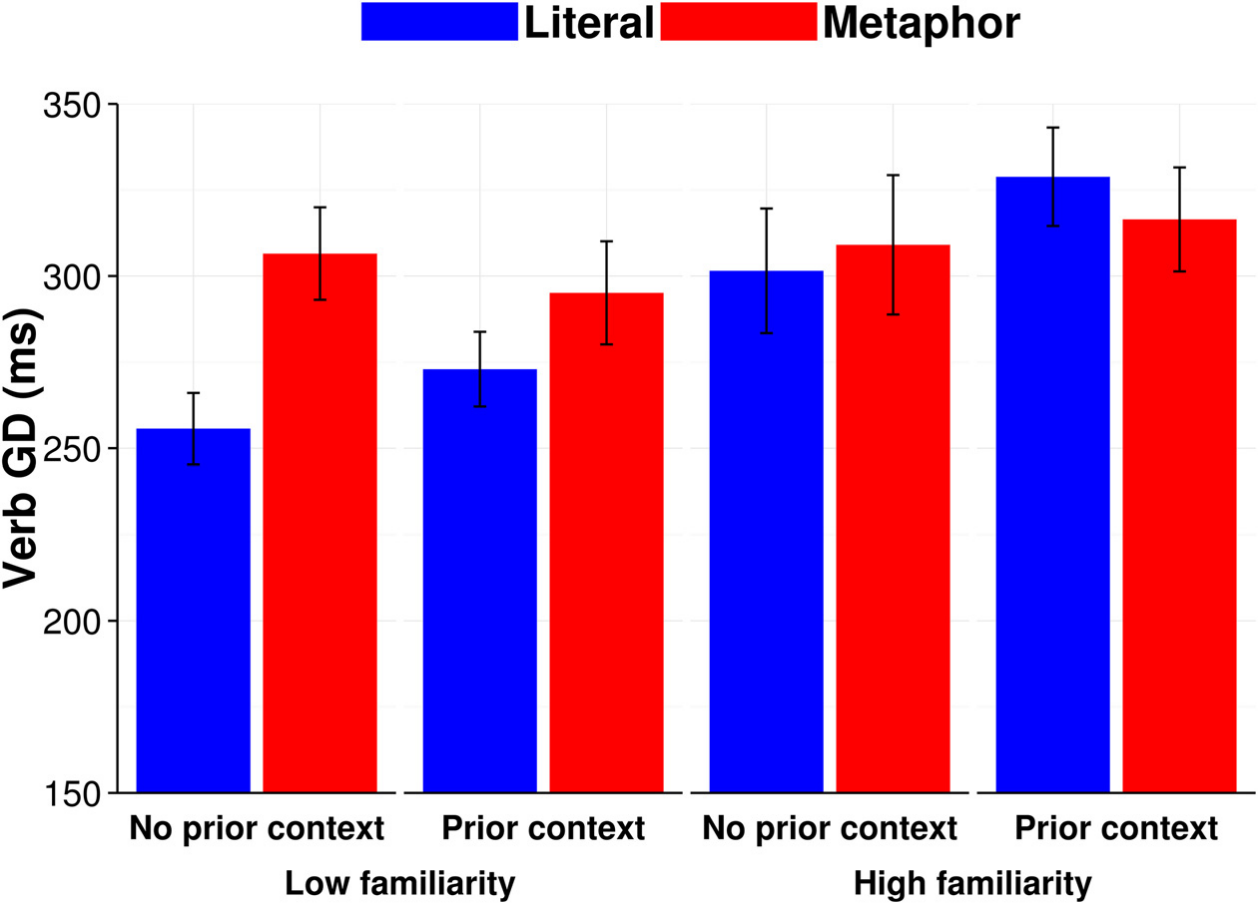
**Context and familiarity subject-averaged (F1) mean reading times (ms) for Verb GD**. Error bars show standard error of the mean.

We removed extreme outliers (Verb GD < 80 ms or > 2000 ms) from the dataset, retaining 94.3% of observations. Stepwise log likelihood model comparisons showed that by-subject and by-item random slopes were not warranted for categorical variables in this model. Subject-averaged (F1) means broken down by metaphor condition, familiarity ratio, and context are presented in Figure 1. The full model is presented in Table 3.

**Table 3:** **Effect sizes (*b*), standard errors (*SE*), and *p*-values for the verb gaze duration logistic LME model**.

**Fixed effects**	**Verb gaze duration**		
	***b***	***SE***	***p***
Condition	0.02	0.02	0.16
Prior context	0.04	0.02	0.01^*^
Familiarity ratio (scaled)	0.03	0.01	0.06
Executive control score (scaled)	0.04	0.03	0.16
Condition^*^Prior context	−0.03	0.03	0.40
Condition^*^Familiarity	−0.04	0.02	0.01^*^
Prior context^*^Familiarity	0.01	0.02	0.56
Condition^*^Executive control	0.00	0.02	0.87
Prior context^*^Executive control	−0.04	0.02	0.02^*^
Familiarity^*^Executive control	0.01	0.01	0.17
Condition^*^Prior context^*^Familiarity	0.01	0.03	0.75
Condition^*^Prior context^*^Executive control	−0.01	0.03	0.70
Condition^*^Familiarity^*^Executive control	0.02	0.02	0.22
Prior context^*^Familiarity^*^Executive control	0.03	0.02	0.15
Condition^*^Prior context^*^Familiarity^*^Exec. Control	−0.01	0.03	0.86
**Control predictors**	***b***	***SE***	***p***
(Intercept)	5.60	0.03	0.00^*^
**Random effects**	**Variance**		
Subject	0.0233		
Item	0.0074		
Residual	0.1405		

* *p ≤ 0.05*.

We found a significant interaction between condition and familiarity ratio (*b* = −0.04, *SE* = 0.02, *p* < 0.05), indicating that verbs used in a metaphorical sentence had longer gaze durations than the same verbs used in a literal sentence to the extent that they were low familiar. To further assess the source and direction of this interaction, we computed sub-models where the data were median split into high and low metaphor-literal familiarity ratios. Readers' Verb GD were longer for low familiar verbs in metaphor sentences (e.g., *The textbook snored on the desk at the end of the day*) compared to low familiar verbs in literal sentences (e.g., *The sailor snored in the hammock at the end of the day*) (*b* = 0.08, *SE* = 0.02, *p* < 0.001); there were no significant effects for high familiar metaphor verbs. Thus, it is likely that readers considered high familiarity ratio verbs to be ambiguous in terms of their metaphoric vs. literal uses, leading to no differences in Verb GD on the verbs as a function of whether they were intended metaphorically or literally.

**Figure 2:**
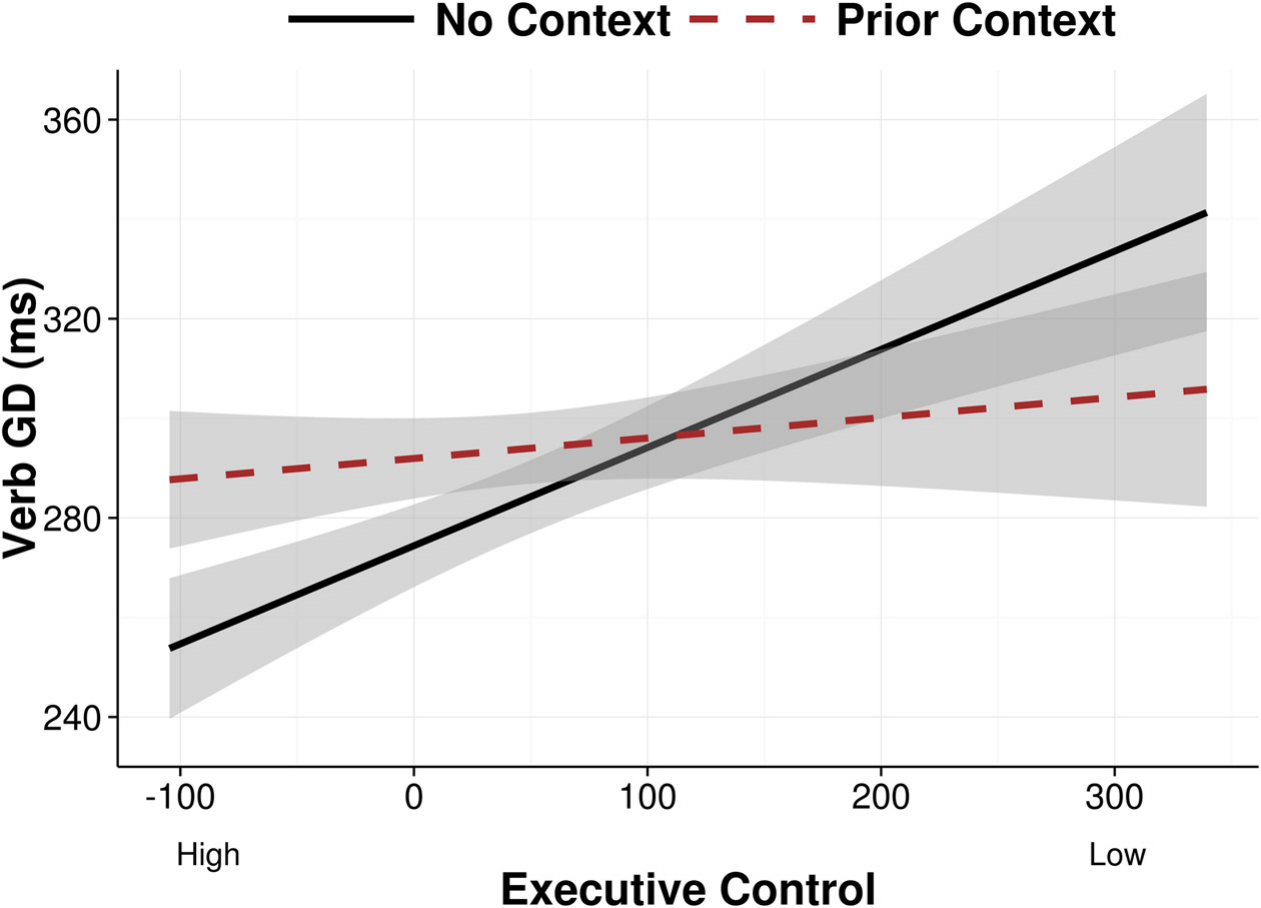
**Verb GD partial effects as a function of prior context and executive control after removing the effects of condition and familiarity, and between-subject and between-item variance**. Error bands show 95% confidence intervals.

We also found an interaction between executive control and presence of a prior context, which did not interact with condition (*b* = −0.04, *SE* = 0.02, *p* < 0.05). This effect indicated that readers with higher executive control read verbs more slowly when there was a prior context, across both metaphorical and literal sentences; In contrast, readers with low executive control showed no difference in Verb GD as a function of prior context (see the partial effects plot in Figure 2). This suggests that readers with high executive control expended more effort to commit to a particular interpretation of the verb at the point of the verb, while readers with low executive control did not.

#### Metaphor TRT

**Figure 3:**
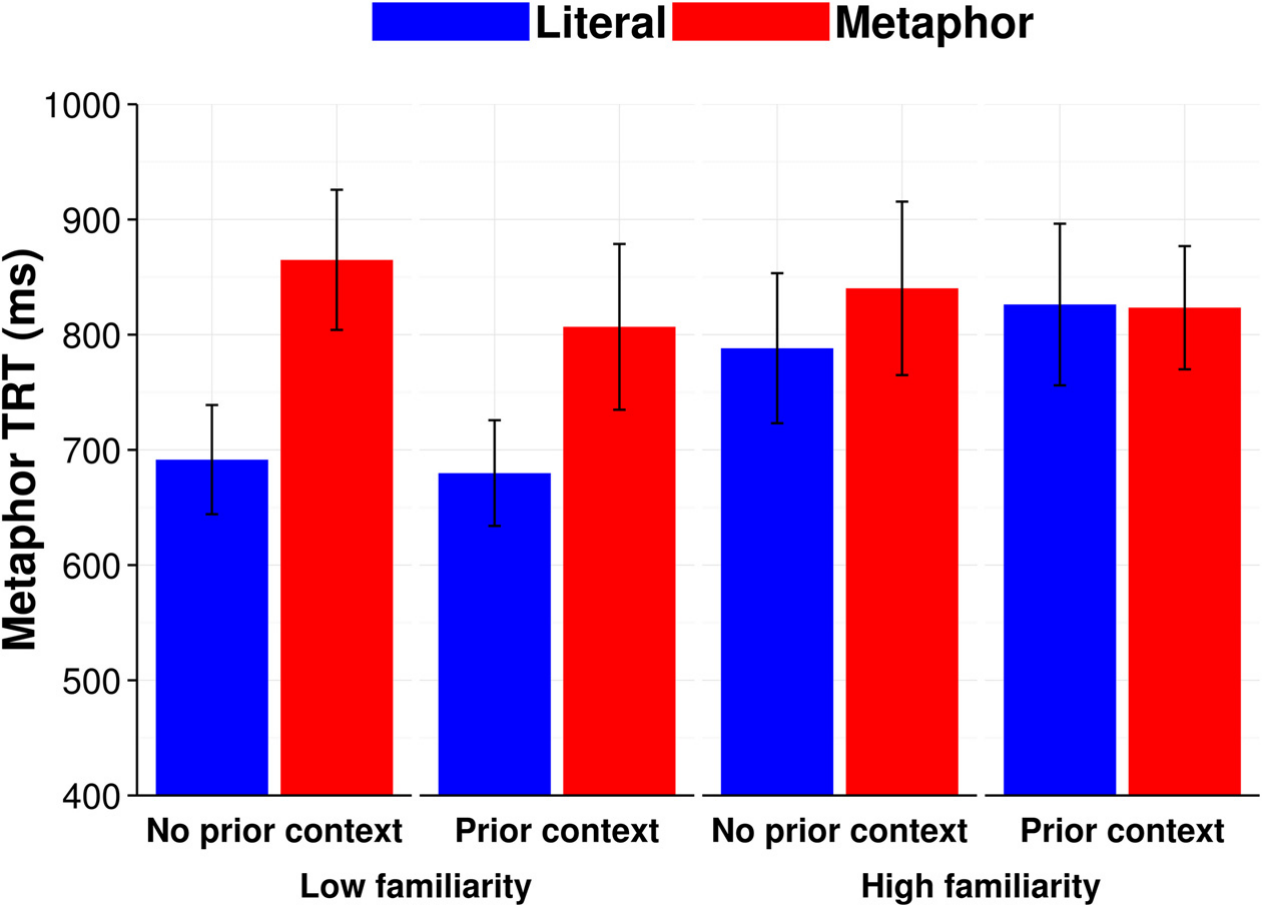
**Context and familiarity subject-averaged (F1) mean reading times (ms) for Metaphor TRT**. Error bars show standard error of the mean.

The models fit to Metaphor TRT included a covariate for character length (continuous) because metaphor length, unlike verb length, varied across sentences with metaphoric vs. literal verb use (e.g., *model flitted* vs. *butterfly flitted*). Extreme outliers were once again removed (Metaphor TRT < 80 ms or > 4000 ms), leaving 94.5% of the observations. Log likelihood model comparisons showed that by-subject and by-item random slopes were warranted for condition and prior context and were thus included. Subject-averaged (F1) means broken down by metaphor condition, familiarity ratio, and context are presented in Figure 3.

**Figure 4:**
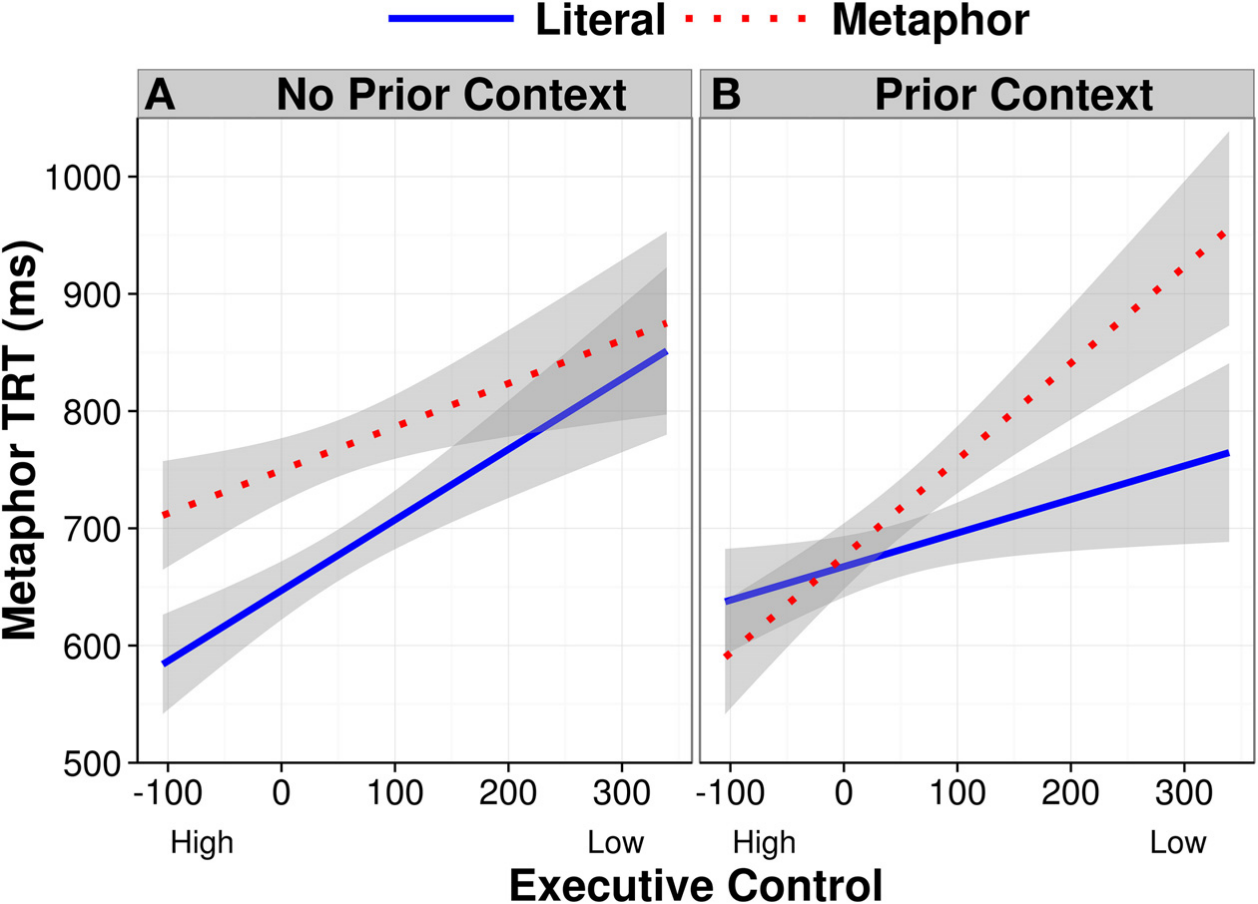
**Metaphor TRT partial effects as a function of condition and executive control in sentences with (A) No Prior Context and with (B) Prior Context after removing the effects of familiarity and noun length, and between-subject and between-item variance**. Error bands show 95% confidence intervals.

As seen in Table 4, there was a two-way interaction between context and condition (*b* = −0.09, *SE* = 0.05, *p* = 0.05), and a three-way interaction between condition, context, and executive control (*b* = 0.08, *SE* = 0.04, *p* = 0.05; see the partial effects plot in Figure 4). To determine the source of the three-way interaction, we ran sub-models split by trials where there was a prior context and when there was not a prior context. In the model fit to the data without a prior context, there was only a main effect of condition (*b* = 0.13, *SE* = 0.04, *p* < 0.001). In the model fit to the data with a prior context, there was a trend for an interaction between condition and executive control which did not reach significance (*b* = 0.04, *SE* = 0.03, *p* = 0.11). In addition to this sub-model, we also ran sub-models split by condition (i.e., metaphor or literal sentences); the model for the metaphor sentences showed a main effect for context (*b* = −0.08, *SE* = 0.04, *p* = 0.05).

**Table 4:** **Effect sizes (*b*), standard errors (*SE*), and *p*-values for the metaphor total reading time logistic LME model**.

**Fixed effects**	**Metaphor total reading time**		
	***b***	***SE***	***p***
Condition	0.09	0.03	0.01^*^
Prior context	−0.03	0.03	0.29
Familiarity ratio (scaled)	−0.01	0.02	0.50
Executive control score (scaled)	0.06	0.05	0.21
Condition^*^Prior context	−0.09	0.05	0.05^*^
Condition^*^Familiarity	−0.04	0.03	0.19
Prior context^*^Familiarity	0.01	0.02	0.82
Condition^*^Executive control	0.01	0.02	0.66
Prior context^*^Executive control	0.00	0.03	0.99
Familiarity^*^Executive control	−0.01	0.01	0.30
Condition^*^Prior context^*^Familiarity	−0.01	0.04	0.74
Condition^*^Prior context^*^Executive control	0.08	0.04	0.05^*^
Condition^*^Familiarity^*^Executive control	0.01	0.02	0.62
Prior context^*^Familiarity^*^Executive control	−0.02	0.02	0.44
Condition^*^Prior context^*^Familiarity^*^Exec. control	−0.03	0.04	0.42
**Control predictors**	***b***	***SE***	***p***
(Intercept)	6.49	0.05	0.00^*^
Noun length (scaled)	0.07	0.02	0.00^*^
**Random effects**	**Variance**		
Subject	0.0917		
Subject|Condition	0.0000		
Subject|Prior context	0.0143		
Subject|Condition|Prior context	0.0222		
Item	0.0187		
Item|Condition	0.0409		
Item|Prior context	0.0097		
Item|Condition|Prior context	0.0186		
Residual	0.1639		

* *p ≤ 0.05*.

Taken together, the trends observed in the follow-up analyses suggest that the best interpretation of the original three-way interaction is that participants with high executive control read metaphors as quickly as literal sentences when there was a prior supportive context. In all other cases, all people read metaphors more slowly than literal sentences. This Metaphor TRT finding is compatible with the one reported above for Verb GD, in that they together suggest that participants with high executive control spent more time reading the verb following a prior context, thus, their efforts toward contextual integration occurred earlier than that found for participants with low executive control.

#### Regression probability into prior context

Given the differences between readers with high and low executive control in Metaphor TRT as a function of prior context, we wished to determine whether and how executive control related to how people read the prior context word itself (e.g., *unopened* in *unopened textbooks snored*). We thus calculated the probability that readers would regress into the prior context word at any point while reading the sentence, and analyzed these data using a generalized LME model. As the model only evaluates data from sentences that had prior contexts, it only included a three-way interaction term for condition ^*^executive control^*^familiarity ratio, unlike the verb and metaphor region models above. Log likelihood model comparisons showed that random slopes were not warranted in this model.

**Figure 5:**
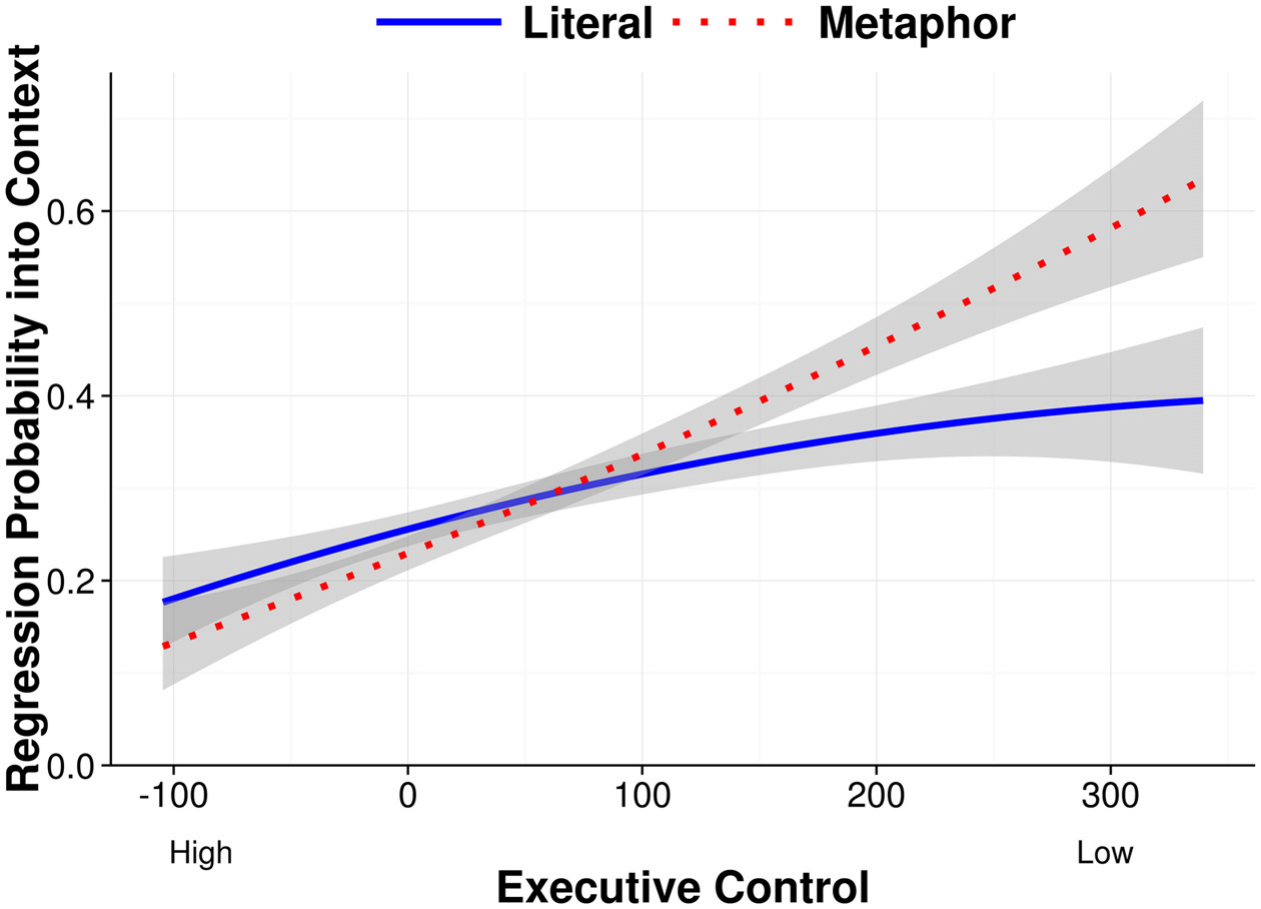
**Regression Probability into Prior Context partial effects as a function of condition and executive control after removing the effects of familiarity and adjective length, and between-subject and between-item variance**. Error bands show 95% confidence intervals.

As seen in Table 5, and consistent with the idea that low executive control readers semantically committed to a particular context-driven interpretation of the verb after the first pass, we found a significant interaction for condition^*^executive control (*b* = 0.31, *SE* = 0.16, *p* = 0.05). This interaction indicated that readers with low executive control had a considerably higher probability of regressing into the prior context, and particularly when they were reading a metaphor sentence rather than a literal sentence (i.e., *textbooks snored* rather than *sailors snored*; see the partial effects plot in Figure 5).

**Table 5:** **Effect sizes (*b*), standard errors (*SE*), and *p*-values for the regression into prior context generalized LME model**.

**Fixed effects**	**Regression probability into prior context**		
	***b***	***SE***	***p***
Condition	−0.03	0.15	0.85
Familiarity ratio	0.12	0.11	0.25
Executive control score (scaled)	0.40	0.19	0.03^*^
Condition^*^Familiarity	−0.13	0.17	0.44
Condition^*^Executive control	0.31	0.16	0.05^*^
Familiarity^*^Executive control	0.06	0.09	0.54
Condition^*^Familiarity^*^Executive control	−0.13	0.18	0.47
**Control predictors**	***b***	***SE***	***p***
(Intercept)	−1.08	0.20	0.00^*^
Context length (scaled)	0.01	0.09	0.89
**Random effects**	**Variance**		
Subject	1.0436		
Item	0.2040		
Residual	*n/a*		

* *p ≤ 0.05*.

### Discussion

In Experiment 1, we examined whether domain-general executive control related to how people read verbs used metaphorically or literally as a function of familiarity and prior context. We found that readers with high but not low executive control took the prior context into account at the point of the verb on the first pass: They exhibited longer Verb GD when a prior context occurred, irrespective of metaphor familiarity. Interestingly, although familiarity speeded Verb GD generally, this general facilitative effect of familiarity did not interact with prior context or executive control.

With respect to later reading measures, however, executive control did interact with condition and context. In terms of Metaphor TRT, people with high executive control showed longer reading times for metaphorical vs. literally intended verbs when there was a prior context. In contrast, people with low executive control did not show this difference, rather they were slower across the board for metaphors. Readers with low executive control were also more likely than those with high executive control to regress back into the context word, suggesting that they had to work harder to make sense of the sentence after it had been fully read and the intended meaning became clear. Thus, when the context biased a particular interpretation (e.g., *unopened textbooks snored*), people with high but not low executive control spent more time reading the metaphorical verb, presumably to semantically commit to the contextually appropriate interpretation on the first pass. Further, they spent less time rereading both the metaphorical regions of the sentence (e.g., *textbooks snored*) or regressing to the biased context word (e.g., *unopened*). Consequently, readers with high executive control displayed a more efficient reading strategy by integrating contextual cues as they occurred on the first pass, whereas readers with low executive control were less likely to do so.

While the overall pattern of metaphor data is relatively clear, one open question is whether a similar pattern of executive function interactions occurs for other forms of figurative language, such as idiomatic expressions, that have fewer on-line comprehension demands. Idiomatic expressions, like metaphors, have figurative meanings that can be more or less familiar (e.g., *kick the bucket*, which is familiar in English and figuratively means “to die”; *bore his cross*, which is known but less familiar in English and figuratively means to “accept one's burden in life”). However, unlike the metaphors used in Experiment 1, the component words of idioms have a high likelihood of co-occurring, independent of what meaning is intended (Wulff, 2008). The implication of this difference between idioms and metaphors is that encountering the initial words of an idiom (e.g., *kick the…*) may enable people to strongly anticipate their completion (e.g., *bucket*), particularly when idioms are highly familiar. This early anticipation of idiom-final words might in turn enable a head start on semantic processing (Cacciari and Tabossi, 1988; Titone and Connine, 1994), such that the interpretive demands faced when one ultimately encounters an idiom-final word are reduced. In this way, idioms might differ from the situation engendered by metaphors (particularly those included in Experiment 1), where there is no basis upon which to anticipate a figuratively biased verb at a lexical level (e.g., *The unopened textbooks snored*), even in the condition where the context is semantically consistent with a metaphorical interpretation of the verb.

Thus, in Experiment 2, we examined whether the presumed lexical boost afforded idioms compared to metaphors would reduce the overall demands of comprehension, and result in a pattern of data where the same participants in Experiment 1, who showed executive control dependencies for metaphors, would fail to show such dependencies for idioms randomly interspersed in the same experimental set. Of note, because the set of idiomatic sentences included in Experiment 2 were not initially intended to serve this purpose, they are not perfectly comparable in a point for point sense, thus it is not readily possible to statistically compare differences across Experiments 1 and 2. However, qualitative comparison of data patterns across Experiments 1 and 2 may be useful for informing future research efforts that directly compare metaphors to idioms using methods and materials deliberately intended to do so.

## Experiment 2: idiom processing and executive control

### Method

#### Participants

The participants were the same individuals who completed Experiment 1.

#### Stimuli

We created sentences containing idioms that all had the same verb-determiner-noun structure: Subject noun, verb, determiner, object noun, and disambiguating context (*Roxy bit her lip and tried to keep the plans for the surprise party a secret*). Like Experiment 1, we had two regions of interest, Idiom-final noun GD and Idiom TRT, the former reflecting early or first-pass comprehension, and the latter reflecting later or second-pass comprehension.

Each sentence contained an idiom or matched literal phrase, followed by a disambiguating context, which forced a particular interpretation of the idiom. There were three conditions, as seen in Table 6 below. In one condition, idioms were followed by a context that biased the idiom's figurative meaning (Id-Id). In a second condition, idioms were followed by a context that biased the idiom's literal meaning (Id-Lit). In the control condition, a matched literal phrase was always followed by a literal context (Lit-Lit). The stimulus set consisted of 54 idioms that were selected from a larger set of well-normed idioms (Libben and Titone, 2008), which included familiarity ratings on 5-point scale (1 = *I never or almost never encounter the idiom*, and 5 = *I encounter the idiom frequently*).

**Table 6:** **Example low and high familiar sentences from idiom-idiom, idiom-literal, and literal-literal conditions**.

**Condition**	**Sentence**
Idiom phrase in idiomatic context—Low familiar	*Josh bore his cross the entire flight and didn't complain about the snoring man*
Idiom phrase in literal context—Low familiar	*Josh bore his cross down the center aisle of the church during the passion play*
Matched literal phrase in literal context	*Josh lost his cross when he dropped it in the grass on the way home from church*
Idiom phrase in idiomatic context—High familiar	*Roxy bit her lip and tried to keep the plans for the surprise party a secret*
Idiom phrase in literal context—High familiar	*Roxy bit her lip as she rushed through breakfast in a hurry to get to school*
Matched literal phrase in literal context	*Roxy cut her lip on a branch when she climbed too high up the cedar tree*

#### Apparatus

Same as Experiment 1.

#### Procedure

The procedure was identical to Experiment 1 because the idiom sentences analyzed here were randomly intermixed in the metaphor set reported in Experiment 1. For the idiom sentences, participants viewed one of six counterbalanced lists. There were 54 target sentences in each list. An idiom or its literal control, but not both, appeared once in a given list in only one condition. Thus, if a participant viewed the idiom *Roxy bit her lip and tried to keep the plans for the surprise party a secret* in the Id-Id condition, s/he would not see that idiom in the Id-Lit condition (*Roxy bit her lip as she rushed through breakfast in a hurry to get to school*), or its matched literal control in the Lit-Lit condition (*Roxy cut her lip as she rushed through breakfast in a hurry to get to school*). No participant saw any sentence more than once.

### Results

Overall comprehension accuracy was 94.5%, indicating that participants were attentive during the experiment.

The same fixed effect structure was applied to each eye movement measure (i.e., Noun GD and Idiom TRT). The fixed effect structure included a three-way interaction term for familiarity (continuous)^*^executive control (continuous)^*^condition (Id-Id, Id-Lit or Lit-Lit; deviation coded). As in Experiment 1, the continuous predictors were scaled, maximum correlations (all < 0.28) showed minimal effects of collinearity, and random intercepts were included for subjects and items. For ease of data interpretation, we present the means and standard deviations for all continuous factors in Table 7, with familiarity categorized into high and low with a median split, and the executive control means and standard deviations repeated from Experiment 1.

**Table 7:** **Means and standard deviations for familiarity and executive function in Experiment 2, split by median**.

**Variable**	**Mean**	**Min**	**Max**	**SD**
Low familiarity (item rating)	2.67	1.67	3.23	0.45
High familiarity (item rating)	4. 08	3.37	4.80	0.39
Low executive control (cost score in ms)	119	35	339	83
High executive control (cost score in ms)	−19	−105	31	41

#### Noun GD

There were no significant interactions or main effects of idiomatic condition. Thus, idiom-final words were read equally fast in all experimental conditions. As well, there were no interactions with executive control.

#### Idiom TRT

**Figure 6:**
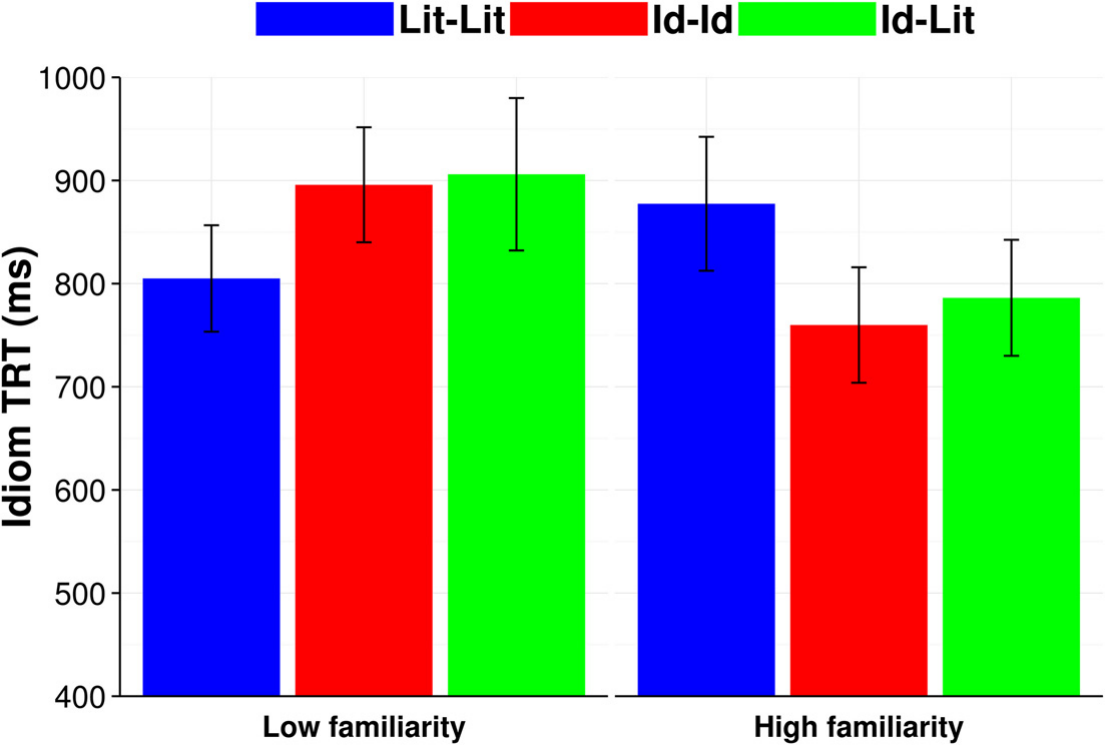
**Familiarity subject-averaged (F1) mean reading times (ms) for Idiom TRT**. Error bars equal standard error of the mean.

A covariate was added for idiom length. We removed extreme outliers (Idiom TRT < 80 ms or > 4000 ms), retaining 95.13% of the total observations. Log likelihood model comparisons showed that by-subject and by-item random slopes were supported for condition in the model. Subject-averaged (F1) means broken down by condition and familiarity are presented in Figure 6.

**Figure 7:**
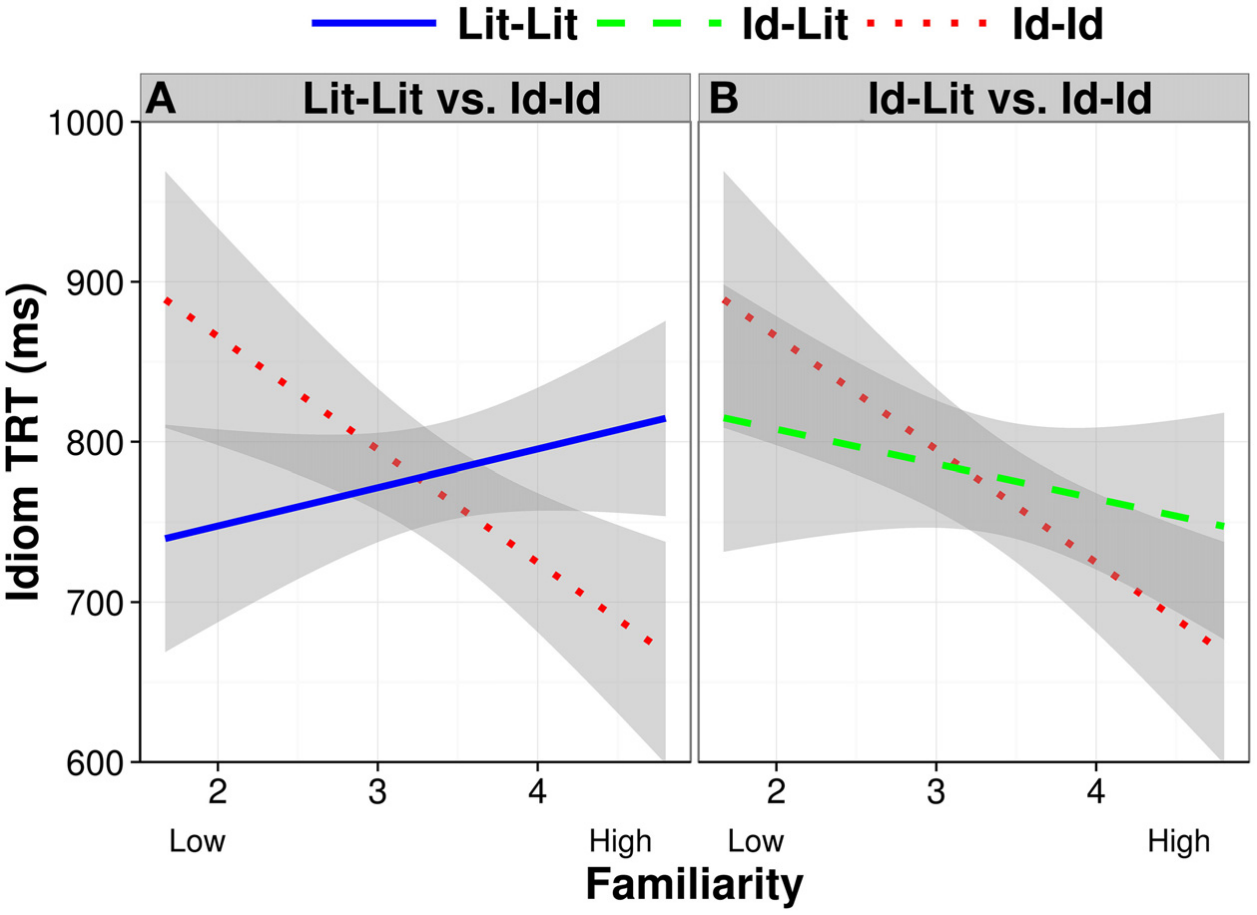
**Idiom TRT partial effects as a function of condition and familiarity after removing the effects of idiom length and executive control**. Error bands show 95% confidence intervals. Id-Id is separately contrasted with Lit-Lit in **(A)** and with Id-Lit in **(B)** so that the difference between Id-Id and Lit-Lit is clear, which is otherwise masked by the error bands for Id-Lit (There was no difference between Lit-Lit and Id-Lit).

As seen in Table 8, we found a significant interaction of condition by familiarity (*b* = −0.12, *SE* = 0.04, *p* < 0.05), indicating that readers had shorter total reading times for high familiar Id-Id phrases (e.g., *Roxy bit her lip and tried to keep the plans for the surprise party a secret*) compared to their reading times for low familiar Id-Id phrases (e.g., *Josh bore his cross the entire flight and didn't complain about the snoring man*) (see the partial effects plot in Figure 7). There was no such interaction with familiarity for the Id-Lit contrast in this model. Moreover, a treatment coded model with Lit-Lit as the baseline showed an interaction with familiarity for Lit-Lit vs. Id-Id (*b* = −0.12, *SE* = 0.04, *p* < 0.05), but not Lit-Lit vs. Id-Lit (*p* > 0.20), but a relevelled model with Id-Id as the baseline showed a trend for an interaction with familiarity for Id-Id vs. Id-Lit (*b* = 0.06, *SE* = 0.03, *p* = 0.06). These interactions are shown in Figure 7. No other effect was significant.

**Table 8:** **Effect sizes (*b*), standard errors (*SE*), and *Pr* (>|*t*|) values for the idiom total Reading time logistic LME model**.

**Fixed effects**	**Idiom total reading**		
	**time**		
	***b***	***SE***	***p***
Condition (Id-Id)	−0.03	0.05	0.54
Condition (Id-Lit)	0.00	0.05	0.95
Familiarity (scaled)	−0.03	0.02	0.22
Executive control score (scaled)	0.05	0.05	0.30
Condition (Id-Id)^*^Familiarity	−0.12	0.04	0.01^*^
Condition (Id-Lit)^*^Familiarity	0.01	0.04	0.87
Condition (Id-Id)^*^Executive control	0.06	0.05	0.20
Condition (Id-Lit)^*^Executive control	−0.03	0.04	0.49
Familiarity^*^Executive control	−0.01	0.01	0.37
Condition (Id-Id)^*^Familiarity^*^Exec. control	0.00	0.04	0.96
Condition (Id-Lit)^*^Familiarity^*^Exec. control	−0.03	0.04	0.38
**Control predictors**	***b***	***SE***	***p***
(Intercept)	6.58	0.05	0.00^*^
Idiom length (scaled)	0.07	0.02	0.00^*^
**Random effects**	**Variance**		
Subject	0.0879		
Subject|Condition (Id-Id)	0.0310		
Subject|Condition (Id-Lit)	0.0147		
Item	0.0155		
Item|Condition (Id-Id)	0.0225		
Item|Condition (Id-Lit)	0.0189		
Residual	0.1604		

* *p ≤ 0.05*.

To better locate the source of the familiarity interactions, we computed treatment coded sub-models split into low and high familiarity sentences with Id-Id as the baseline to compare Id-Id vs. Id-Lit and Lit-Lit (since the significant differences in the preceding analyses only involved Id-Id). The model fit to high familiar phrases showed that readers had faster Idiom TRT for Id-Id sentences than for Lit-Lit sentences (*b* = 0.16, *SE* = 0.06, *p* < 0.05). No other effects were significant. Thus, of note, there were no interactions with executive control.

### Discussion

Experiment 2 investigated whether idiom processing was modulated by individual differences in executive control for the same participants tested in Experiment 1. Our results show that high familiar idioms had shorter total reading times than matched literal phrases when the sentence was ultimately biased toward an idiomatic interpretation. Finally, of relevance to our question of interest, individual differences in executive control never interacted with any reading measure.

## General discussion

We used eye movement measures of sentence reading to determine whether familiarity and context modulates metaphor processing as a function of individual differences in executive control. We also assessed how the relationship between individual differences in executive control and comprehension extended qualitatively to idiom processing for the same participants. There were three key findings.

The first key finding was that, in Experiment 1, relative familiarity of a metaphorical vs. literal interpretation of the verb modulated how much time people spent reading the verb on the first pass (see Figure 1). Of note, this effect occurred irrespective of prior context or individual differences in executive control. Accordingly, when metaphor familiarity was low, Verb GD was slower for verbs intended metaphorically than for verbs intended literally. This difference decreased as metaphor familiarity increased (relative to familiarity of the verb's literal interpretation). This suggests that when people encounter verbs intended metaphorically, immediate comprehension is slowed if the metaphorical meaning of the verb is less familiar. The slowing of immediate comprehension potentially reflects some combination of the time necessary for inhibiting the more familiar literal interpretation of the verb, and for generating or retrieving from memory the verb's metaphorical sense.

Our second key finding involved metaphors and executive control in Experiment 1. Specifically, when people encountered a metaphorically or literally intended verb following a prior context that supported whichever interpretation of that verb, readers with high executive control spent more time fixating the verb on the first pass, presumably to immediately integrate the appropriate meaning with the prior context. In contrast, readers with low executive control did not spend extra time fixating the verb under the same circumstances. They consequently experienced comprehension difficulty later on in the sentence, as indicated by longer total reading times of the metaphor region, and a higher likelihood of regressing back to the context word, particularly in the metaphorically biased condition. Thus, these results suggest that high and low executive control readers differed in the rapidity with which they used context to interpret the verb on the first pass, and this difference propagated to later portions of the sentence: High executive control readers made immediate semantic commitments, whereas low executive control readers delayed their semantic commitments pending subsequent disambiguating parts of the sentence (see also Frazier and Rayner, 1990; Pickering and Frisson, 2001, for related work on single-word lexical ambiguity).

Our third key finding involves the role of executive control in idiom processing (Experiment 2). Unlike the global pattern found for metaphor processing, individual differences in executive control did not modulate reading times for idioms in Experiment 2, despite the fact that idiom reading times were affected by idiom familiarity (albeit measured in a very different way than it was for metaphors in Experiment 1). Specifically, high familiar idioms in sentences that had a subsequent idiom context had shorter total reading times for the idiom region than both matched controls and low familiar idioms. This suggests that when an idiom was familiar, people were more likely to entertain its figurative meaning and consequently were less likely to revisit the idiom on the second pass, presumably because the initial semantic commitment made to the figurative interpretation of the phrase was confirmed by subsequent context.

Of note, figurative condition had no significant first-pass effects for gaze duration on idiom-final nouns, unlike the pattern found for metaphor-final verbs, where all readers showed longer gaze durations when there was no prior context and the verb was figuratively intended. This suggests that the global effort needed to resolve a semantic commitment on the first pass is generally reduced for idioms compared to metaphors, perhaps due to the fact that relatively common idiomatic expressions enjoy a lexical boost due to the high co-occurrence of their component words (Wulff, 2008). Thus, it is possible that idioms are so thoroughly lexicalized for native speakers of a language (even ones that are rated as low familiar) that people can partially anticipate their final words and thus get a head start on processing those words lexically and semantically (Cacciari and Tabossi, 1988; Titone and Connine, 1994; Libben and Titone, 2008). This state of affairs may be especially true for natural reading contexts, such as in the current study, where early anticipation of idiom-final words may be enhanced by some amount of parafoveal processing of upcoming words (Kliegl et al., 2007; Hohenstein et al., 2010; Angele et al., 2013). While parafoveal processing of words was also certainly possible for the metaphors in Experiment 1, these metaphors do not likely enjoy the same lexicalized, collocation status as idioms. Thus, readers may not have been as able to extract useful parafoveal information for metaphors that would enable a meaningful head start on processing.

In summary, the results suggest that general cognitive capacities, such as executive control, are important for processing metaphors during natural sentence reading. The results also suggest that not all elements of figurative language may incur the same executive control demands as metaphors. Specifically, executive control demands for idioms during natural reading may differ because idioms are generally more familiar both lexically and semantically compared to metaphorical language. Thus, the results of the present study, while preliminary, suggest that further comparison of metaphors and idioms is a potentially fruitful avenue of inquiry.

## Author contributions

Debra Titone designed the study, Marilena Côté-Lecaldare helped create the materials, Naveed A. Sheikh programmed the experiment, and Marilena Côté-Lecaldare, Katja Häuser and Georgie Columbus acquired the data. Debra Titone, Georgie Columbus, Marilena Côté-Lecaldare and Naveed A. Sheikh analyzed and interpreted the data, and wrote the manuscript. Debra Titone, Georgie Columbus, Naveed A. Sheikh, Shari R. Baum and Katja Häuser revised the final manuscript.

### Conflict of interest statement

The authors declare that the research was conducted in the absence of any commercial or financial relationships that could be construed as a potential conflict of interest.
